# rhG-CSF is associated with an increased risk of metastasis in NSCLC patients following postoperative chemotherapy

**DOI:** 10.1186/s12885-022-09850-4

**Published:** 2022-07-07

**Authors:** Yong Wang, Chen Fang, Renfang Chen, Shangkun Yuan, Lin Chen, Xiaotong Qiu, Xiaoying Qian, Xinwei Zhang, Zhehao Xiao, Qian Wang, Biqi Fu, Xiaoling Song, Yong Li

**Affiliations:** 1 Department of Medical Oncology, The First Affiliated Hospital of Nanchang University, 17 Yongwai Zheng Road, Nanchang, 330000 China; 2 Medical Innovation Center, The First Affiliated Hospital of Nanchang University, 17 Yongwai Zheng Road, Nanchang, 330000 China; 3 Department of Internal Neurology, The Second Affiliated Hospital of Nanchang University, 1 MingDe Road, Nanchang, 330000 China; 4 Department of Rheumatology, The First Affiliated Hospital of Nanchang University, 17 Yongwai Zheng Road, Nanchang, 330000 China; 5 Department of Medical Record Room, The First Affiliated Hospital of Nanchang University, 17 Yongwai Zheng Road, Nanchang, 330000 China

**Keywords:** Recombinant human granulocyte colony-stimulating factor (rhG-CSF), Metastasis, Non-small-cell lung cancer (NSCLC), Distant organ metastasis (DOM), Postoperative chemotherapy

## Abstract

**Background:**

Recombinant human granulocyte colony-stimulating factor (rhG-CSF) reduces neutropenia events and is widely used in cancer patients receiving chemotherapy. However, the effects of rhG-CSF on distant organ metastasis (DOM) in non-small-cell lung cancer (NSCLC) patients following postoperative chemotherapy are not clear.

**Methods:**

A retrospective cohort study was performed on NSCLC patients who underwent complete surgical resection and postoperative systemic chemotherapy at The First Affiliated Hospital of Nanchang University between 1 January 2012 and 31 December 2017. The effect of rhG-CSF on DOM was assessed with other confounding factors using Cox regression analyses.

**Results:**

We identified 307 NSCLC patients who received postoperative systemic chemotherapy (*n* = 246 in the rhG-CSF group, *n* = 61 in the No rhG-CSF group). The incidence of DOM in postoperative NSCLC patients with rhG-CSF treatment was observably higher than in patients without rhG-CSF treatment (48.3% vs. 27.9%, *p* < 0.05). Univariate regression analysis revealed that rhG-CSF and pathological stage were independent risk factors for metastasis-free survival (MFS) (*p* < 0.05). RhG-CSF users had a higher risk of DOM (adjusted HR: 2.33, 95% CI: 1.31–4.15) than nonusers of rhG-CSF. The association between rhG-CSF and the risk of DOM was significant only in patients presenting with myelosuppression (HR: 3.34, 95% CI: 1.86–6.02) and not in patients without myelosuppression (HR: 0.71, 95% CI: 0.17–2.94, Interaction *p*-value*<* 0.01). The risk increased with higher dose density of rhG-CSF compared to rhG-CSF versus no users (*p* for trend< 0.001).

**Conclusion:**

These analyses indicate that rhG-CSF use is related to DOM following postoperative chemotherapy in NSCLC.

## Background

Non-small-cell lung cancer (NSCLC) is one of the most common cancers and the leading cause of cancer death worldwide [1]. Surgery and postoperative adjuvant chemotherapy are the main and standard treatments for early-stage NSCLC. However, the emergence of distant organ metastasis (DOM) is the primary reason for cancer treatment failure and tumour-associated death. DOM is a critical risk factor in patient survival prognosis even when undergoing curative surgical resection [2]. The DOM rate of NSCLC positively reaches 50% [3], and the brain, liver, adrenal gland, and bone are generally the distant organs where NSCLC is more likely to metastasize. Over half of NSCLC patients have metastases at diagnosis [4], and tumour metastasis is the primary reason for death in cancer patients [5]. Therefore, appreciation of the risk factors for distant metastasis may facilitate the selection of scientific and reasonable strategies for reducing the risk of metastasis and improving the curative rate and life quality of NSCLC patients.

Recombinant human granulocyte colony-stimulating factor (rhG-CSF) is commonly used for cancer patients following myelosuppressive cytotoxic chemotherapy, and it decreases the risk of potentially fatal infections and hospitalisation associated with febrile neutropenia (FN) and ensures that the dose intensity of chemotherapy improves the overall survival (OS) [6]. However, the cytokine granulocyte colony-stimulating factor (G-CSF) exhibited pro-tumour activities in numerous recent studies [7]. Increasing evidence revealed that the use of exogenous G-CSF promoted cancer metastasis in preclinical cancer models [8, 9]. Kumar et al. [10] found that G-CSF contributed to the increased migration and survival of ovarian cancer cells by targeting the downstream JAK2/STAT3 signalling pathway. G-CSF was associated with invasive and malignant tumour growth in a head and neck squamous cell carcinoma (HNSCC) mouse model [11].

However, both of these effects were eliminated in the presence of G-CSF inhibitors. However, clinical data rarely indicate that rhG-CSF use in cancer patients is related to a high DOM risk. The impact of rhG-CSF treatment in postoperative chemotherapy patients with myelosuppression on cancer metastasis, especially in NSCLC, is not clear. Therefore, we examined the pro-metastatic character of rhG-CSF in a retrospective cohort of NSCLC patients who received chemotherapy after surgery. These data provide experimental evidence to reduce the occurrence of cancer metastasis by pursuing G-CSF.

## Materials and methods

### Study design and population

The Ethics Committee of the First Affiliated Hospital of Nanchang University, China approved this retrospective cohort study, which abided by the rules of the Declaration of Helsinki. We reviewed the digital medical records of 307 consecutive NSCLC patients for pathological stage IB-IIIB (AJCC system 8th edition) who underwent complete surgical resection via lobectomy or greater and systematic lymph node dissection (lymph node dissection was performed depending on tumour location) and received postoperative system chemotherapy at The First Affiliated Hospital of Nanchang University between January 1, 2012 and December 31, 2017. Complete resection was defined as the macroscopic and microscopic absence of residual cancer (R0). Patients were excluded when they had been diagnosed with cancer other than NSCLC before surgery or when they underwent limited resection (segmentectomy or wedge resection), incomplete resection, neoadjuvant chemotherapy/radiotherapy, metastasis occurring before surgery or before receiving the first postoperative chemotherapy, or a follow-up time of fewer than 30 days after surgery. All patient records were anonymised before analyses. Follow-up duration was counted from the day of surgery to the day of DOM diagnosis, last follow-up, or loss to follow-up. The last follow-up date was December 31, 2020.

One research group blinded to the outcome status collected clinical records data of the potential cases, and another research group blinded to the exposure status collected the outcome and follow-up of the case. The first and the corresponding authors were responsible for all data analyses and interpretation.

### Treatment and data collection

Adjuvant chemotherapy was administered after surgery for NSCLC. Adjuvant chemotherapy dose intensity was applied according to the NCCN guidelines. During postoperative treatment, chemotherapy quickly induces myelosuppression, and rhG-CSFs were given to relieve or prevent myelosuppression. rhG-CSFs were administered 24 h after adjuvant chemotherapy. Patients were divided into two groups based on the use or non-use of rhG-CSF. All rhG-CSF users had at least one rhG-CSF prescription. The effects of the dose of rhG-CSF exposure on the postoperative metastasis risk of NSCLC were also analysed. Four subgroups (≤500 μg, 500–1000 μg, 1000–1500 μg and > 1500 μg) were divided based on the cumulative total rhG-CSF dose. Because of the impact of dose use over time, dose density of rhG-CSF on metastasis was used to evaluate the effects of density of rhG-CSF treatment on the outcome variable metastasis. Length of rhG-CSF exposure time (in days) was measured from the first prescription to the last to calculate the dose density of rhG-CSF treatment.

We reviewed medical records to extract data on demographics, clinicopathological characteristics, and treatment histories: age, weight, sex, smoking habit (never smoked or smoked), anamnesis, histological type, grade, and pathological stage. During the postoperative chemotherapy and follow-up period, regimen and number of chemotherapies, the severity of myelosuppression (Common Terminology Criteria for Adverse Events (CTCAE), version 4.03), fever (> 37.5 °C, using axillary mercury thermometers), and antibiotics were collected.

### Study assessments

All selected NSCLC patients achieved disease-free status after surgery. We reviewed clinical notes and checked all routine imaging records of enrolled patients after adjuvant chemotherapy, including ultrasound (US), thoracic radiography, computed tomography (CT), magnetic resonance imaging (MRI), and single-photon emission computed tomography (SPECT) at the time of diagnosis or during follow-ups, to assess their metastatic status. Metastasis-free survival (MFS) was defined as the duration from the day of chest surgery to investigator-assessed radiographic organ or node metastasis. MFS was censored on the last follow-up date if patients were alive and without any evidence of metastasis. Patient status of metastasis 1, 2, and 3 years after surgery was also measured. Patients without documented clinical or radiographic disease date of metastasis were censored at the last follow-up.

### Statistical analyses

Continuous variables are shown as the means ± SDs, and categorical variables are shown as frequencies (percentages). The t-test (normal distribution) or Kruskal-Wallis rank-sum test (abnormal distribution) for continuous variables and *χ*^2^ tests for categorical data were used to analyse each covariate’s required data distribution between the rhG-CSF and No rhG-CSF groups. We used the Kaplan-Meier method to calculate overall MFS rates, and the differences in survival for univariate comparisons were calculated using the log-rank test. Forest plots were used to describe the underlying effect modification by each covariate. Interaction analysis was performed by calculating the respective categorical variable product terms individually in the model. Whether rhG-CSF and other covariates had an independent effect on NSCLC patients’ metastasis following postoperative chemotherapy separately was evaluated using univariate logistic regression and multivariate logistic regression models. The level of significance was established at *p* = 0.05. The statistical software packages R (http://www.R-project.org, The R Foundation) and EmpowerStats (http://www.empowerststs.com, X&Y Solutions, Inc., Boston, MA, USA) were used to perform all statistical analyses.

## Results

### Patients’ baseline characteristics

Of the 307 postoperative NSCLC patients included in the study, 246 (80.1%) patients who received rhG-CSF treatment during chemotherapy were used as the study group (rhG-CSF group), and 61 (19.9%) patients who did not receive rhG-CSF were included in the control group (No rhG-CSF group). The clinical characteristics and baseline parameters of the No rhG-CSF and rhG-CSF groups are presented in Table 1. The mean age (56.52 years vs. 57.74 years) of participants was primarily similar in the No rhG-CSF group and rhG-CSF group. Approximately half the number of patients treated with the taxane+platinum chemotherapy regimen (45.90% vs. 41.06%) and received four cycles of chemotherapy (47.54% vs. 55.69%) after surgery in both groups. Patients with myelosuppression were observed more frequently in the rhG-CSF group than patients without rhG-CSF treatment (56.50% vs. 8.20%, *p* < 0.001). Most patients with myelosuppression after chemotherapy tend to use rhG-CSF in clinical practice. A total of 107 patients received rhG-CSF as primary prophylaxis, and 139 patients received rhG-CSF as secondary prophylaxis. Essential characteristics were comparable between the No rhG-CSF and rhG-CSF groups. Patients in the two groups were similar in age, weight, sex, smoking, histology, differentiation, pathological stage, chemotherapy, fever, antibiotics, and complications. We excluded patients with a poor general physical condition before surgery and chemotherapy treatment. Therefore, the Eastern Cooperative Oncology Group performance status (ECOG PS) level of all postoperative NSCLC patients was 0–1, and there was no noticeable difference between the rhG-CSF and No rhG-CSF groups.

**Table 1:** Patient characteristics included in the study

Feature	No rhG-CSF (***n*** = 61)	rhG-CSF (***n*** = 246)	***P*** value
Age, y	56.52 ± 10.22	57.74 ± 8.13	0.25
Weight, Kg	60.85 ± 10.80	59.40 ± 9.51	0.30
Gender, n (%)			0.97
Male	46 (75.41%)	186 (75.61%)	
Female	15 (24.59%)	60 (24.39%)	
Smoking, n (%)			0.96
Never	24 (39.34%)	96 (39.02%)	
Former/current	37 (60.66%)	150 (60.98%)	
Histopathology, n (%)			0.96
Adenocarcinoma	29 (47.54%)	122 (49.59%)	
Squamous carcinoma	29 (47.54%)	112 (45.53%)	
Others^a^	3 (4.92%)	12 (4.88%)	
Differentiation, n (%)			0.32
I	2 (3.28%)	7 (2.85%)	
II	7 (11.48%)	52 (21.14%)	
III	7 (11.48%)	33 (13.41%)	
NA	45 (73.77%)	154 (62.60%)	
Clinical stage, n (%)			0.152
IB	17 (27.87%)	43 (17.48%)	
IIA	2 (3.28%)	14 (5.69%)	
IIB	28 (45.90%)	97 (39.43%)	
IIIA	12 (19.67%)	78 (31.71%)	
IIIB	2 (3.28%)	14 (5.69%)	
Chemotherapy, n (%)			0.24
Taxane+Platinum	28 (45.90%)	101 (41.06%)	
Gemcitabine+Platinum	10 (16.39%)	55 (22.36%)	
Pemetrexed+Platinum	21 (34.43%)	87 (35.37%)	
Taxane	1 (1.64%)	1 (0.41%)	
Pemetrexed	1 (1.64%)	0 (0.00%)	
Others^b^	0 (0.00%)	2 (0.81%)	
No. of chemotherapy, n (%)			0.01
< 4	28 (45.90%)	72 (29.27%)	
≥ 4	33 (54.10%)	174 (70.73%)	
Myelosuppression, n (%)			< 0.001
0	56 (91.80%)	107 (43.50%)	
1	2 (3.28%)	55 (22.36%)	
2	2 (3.28%)	49 (19.92%)	
3	0 (0.00%)	20 (8.13%)	
4	1 (1.64%)	15 (6.10%)	
Fever, n (%)			0.30
No	58 (95.08%)	224 (91.06%)	
Yes	3 (4.92%)	22 (8.94%)	
Antibiotic, n (%)			0.64
No	56 (91.80%)	221 (89.84%)	
Yes	5 (8.20%)	25 (10.16%)	
Complications, n (%)			0.90
No	40 (65.57%)	167 (67.89%)	
Hypertension	9 (14.75%)	31 (12.60%)	
Tuberculosis	2 (3.28%)	5 (2.03%)	
Gastritis/Ulcers	3 (4.92%)	14 (5.69%)	
Hepatitis	0 (0.00%)	9 (3.66%)	
Diabetes	1 (1.64%)	5 (2.03%)	
Gallbladder disease	2 (3.28%)	3 (1.22%)	
Enteropatia	1 (1.64%)	2 (0.81%)	
Thyroid disease	1 (1.64%)	3 (1.22%)	
Coronary heart disease	1 (1.64%)	2 (0.81%)	
Chronic bronchitis/COPD	1 (1.64%)	5 (2.03%)	

Values are n (%) or mean ± SD

*No.* Number, *NA* Not available, *rhG-CSF* Recombinant human granulocyte-colony stimulating factor, *COPD* Chronic obstructive pulmonary disease

^a^Others: large cell neuroendocrine carcinoma, atypical carcinoid, adenosquamous carcinoma, lymphoepithelioma-like carcinoma, large cell carcinoma, pulmonary blastoma

^b^Others: Irinotecan+Platinum, Etoposide+Platinum

### Prognostic factors for metastasis

A total of 136 of the 307 postoperative NSCLC patients (44.30%) had distant organ metastases following the chest surgery over the median follow-up period of 33.63 months. A total of 119 (48.3%) patients were in the rhG-CSF group, and 17 (27.9%) patients were in the No rhG-CSF group, which suggests that the DOM rate was significantly higher in the study group than the control group (*p* < 0.05). RhG-CSF treatment significantly correlated with metastasis in univariate Cox regression analysis (HR 2.30, 95% CI 1.36–3.88, *p* < 0.01). We found that the different doses and density gradients of rhG-CSF likely played an essential role in distant organ metastases (Table 2). Pathological stage was also associated with metastasis following NSCLC postoperative chemotherapy (Stage IIA: HR 1.01, 95% CI 0.37–2.78, *p* = 0.99; Stage IIB: HR 1.90, 95% CI 1.10–3.50, *p* = 0.02; Stage IIIA: HR 3.52, 95% CI 1.97–6.27, *p* < 0.01; Stage IIIB: HR 4.39, 95% CI 1.96–9.81, *p* < 0.01), which is consistent with previous studies [12].

**Table 2:** Effects of risk factors on metastasis following operation by univariate analysis

Variables	n (%)	HR (95%CI)	***P*** value
Histopathology			
Others^a^	15 (4.89%)	1	
Adenocarcinoma	151 (49.19%)	1.20 (0.52, 2.75)	0.68
Squamous carcinoma	141 (45.93%)	0.95 (0.41, 2.23)	0.91
Differentiation			
I	9 (2.93%)	1	
II	59 (19.22%)	2.00 (0.60, 6.63)	0.26
III	40 (13.03%)	0.95 (0.26, 3.46)	0.94
NA	199 (64.82%)	1.74 (0.55, 5.51)	0.34
Complications			
No	207 (67.43%)	1	
Yes	100 (32.57%)	0.69 (0.47, 1.01)	0.06
Clinical stage			
IB	60 (19.54%)	1	
IIA	16 (5.21%)	1.01 (0.37, 2.78)	0.99
IIB	125 (40.72%)	1.90 (1.10, 3.50)	0.02
IIIA	90 (29.32%)	3.52 (1.97, 6.27)	< 0.01
IIIB	16 (5.21%)	4.39 (1.96, 9.81)	< 0.01
Chemotherapy			
Taxane+Platinum	129 (42.02%)	1	
Gemcitabine+Platinum	65 (21.17%)	1.08 (0.67, 1.72)	0.76
Pemetrexed+Platinum	108 (35.18%)	1.07 (0.73, 1.57)	0.72
No. of chemotherapy			
< 4	100 (32.57%)	1	
≥ 4	207 (67.43%)	0.71 (0.50, 1.02)	0.06
Myelosuppression			
No	163 (53.09%)	1	
Yes	144 (46.91%)	0.93 (0.66, 1.31)	0.69
Fever			
No	282 (91.86%)	1	
Yes	25 (8.14%)	1.47 (0.81, 2.67)	0.21
Antibiotic			
No	277 (90.23%)	1	
Yes	30 (9.77%)	1.34 (0.77, 2.35)	0.30
rhG-CSF			
No rhG-CSF	61 (19.87%)	1	
rhG-CSF	246 (80.13%)	2.30 (1.36, 3.88)	< 0.01
Dosage (μg)			
0	61 (19.87%)	1	
≤ 500	96 (31.27%)	2.67 (1.52, 4.70)	< 0.01
500–1000	85 (27.69%)	2.24 (1.25, 4.02)	< 0.01
1000–1500	32 (10.42%)	1.63 (0.80, 3.35)	0.18
> 1500	33 (10.75%)	2.29 (1.15, 4.57)	0.02
Dosage density (μg/day)			
0	67 (21.82%)	1	
< 15	71 (23.13%)	1.60 (0.91, 2.82)	0.10
15–60	89 (28.99%)	1.76 (1.01, 3.06)	0.04
60–200	38 (12.38%)	2.45 (1.30, 4.60)	0.01
≥ 200	42 (13.68%)	3.89 (2.12, 7.12)	< 0.01

*HR* Hazard ratio, *CI* Confidence interval, *rhG-CSF* Recombinant human granulocyte-colony stimulating factor, *No.* Number, *NA* Not available

^a^Others: large cell neuroendocrine carcinoma, atypical carcinoid, adenosquamous carcinoma, lymphoepithelioma-like carcinoma, large cell carcinoma, pulmonary blastoma

### Impact of rhG-CSF treatment on the risk of metastasis

**Fig. 1:**
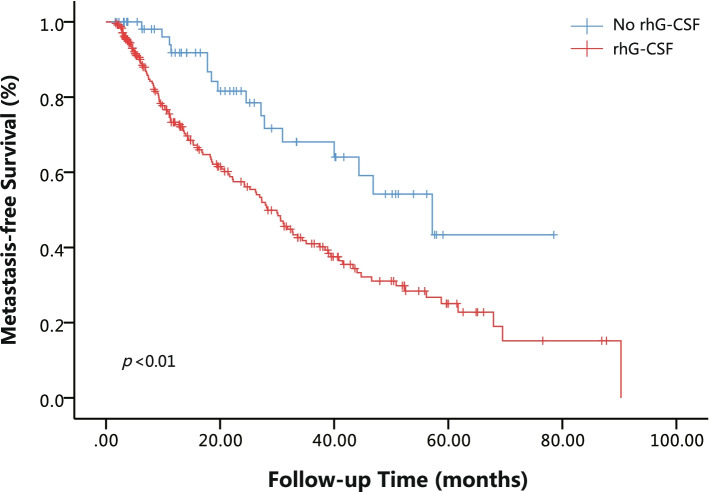
Kaplan-Meier curves of metastasis-free survival (MFS) for postoperative NSCLC patients accepted chemotherapy stratified by No rhG-CSF and rhG-CSF

**Fig. 2:**
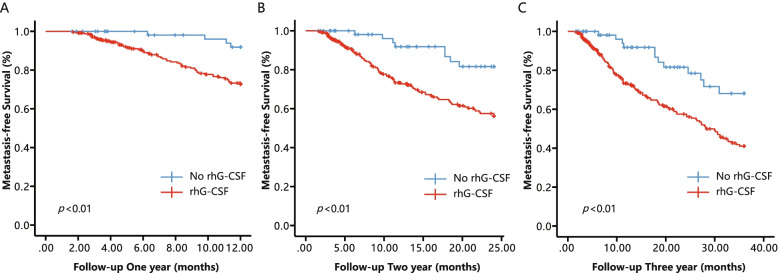
Kaplan-Meier curves of metastasis-free survival (MFS) for postoperative NSCLC patients accepted chemotherapy during different follow-up times stratified by No rhG-CSF and rhG-CSF. **A**-**C** Follow-up postoperative NSCLC patients accepted chemotherapy 1, 2, and 3 years

The Kaplan-Meier curve of the metastasis-free survival rates of postoperative NSCLC patients is shown in Fig. 1. The risk of DOM was substantially increased in the rhG-CSF group compared to the No rhG-CSF group. RhG-CSF treatment was also associated with poorer MFS at 1 year (HR 4.00, 95% CI 1.45–11.05, *p =* 0.007), 2 years (HR 3.11, 95% CI 1.50–6.43, *p =* 0.002) and 3 years (HR 2.59, 95% CI 1.42–4.71, *p* = 0.02) of postoperative follow-up (Fig. 2).

Accounting for other vital covariates, Table 3 further quantifies the efficacy of rhG-CSF in promoting metastasis in multivariate Cox regression analysis adjusted for some or all the confounders. For patients with rhG-CSF treatment, the DOM incidence was increased from 27.8 to 48.3% in postoperative NSCLC patients compared to the No rhG-CSF group, which represents a relative risk increase of 1.41 times in the adjusted I model (HR 2.41, 95% CI 1.42–4.11, *p* < 0.01) and 1.33 times in the adjusted II model (HR 2.33, 95% CI 1.31–4.15, *p* < 0.01). We performed similar analyses for postoperative follow-up at 1, 2, and 3 years, and rhG-CSF treatment was associated with an increased risk of metastasis in the postoperative NSCLC patients (Table 4). RhG-CSF was an independent adverse factor of DOM.

**Table 3:** Effect of any rhG-CSF use on metastasis following operation in NSCLC

	Total, n	Events, n (%)	Non-adjusted		Adjusted I		Adjusted II	
			HR (95% CI)	***p***	HR (95% CI)	***p***	HR (95% CI)	***p***
**rhG-CSF**								
No rhG-CSF	61	16 (26.23%)	1		1		1	
rhG-CSF	246	119 (48.37%)	2.30 (1.36, 3.88)	< 0.01	2.41 (1.42, 4.11)	< 0.01	2.33 (1.31, 4.15)	< 0.01
**Dosage (μg)**								
0	61	16 (26.23%)	1		1		1	
≤ 500	96	49 (51.04%)	2.67 (1.52, 4.70)	< 0.01	2.86 (1.61, 5.08)	< 0.01	2.74 (1.50, 5.02)	< 0.01
500–1000	85	38 (44.71%)	2.24 (1.25, 4.02)	< 0.01	2.32 (1.28, 4.18)	< 0.01	2.30 (1.20, 4.43)	0.01
1000–1500	32	14 (43.75%)	1.63 (0.80, 3.35)	0.18	1.68 (0.81, 3.50)	0.16	1.48 (0.69, 3.18)	0.32
> 1500	33	18 (54.55%)	2.29 (1.15, 4.57)	0.02	2.41 (1.20, 4.84)	0.01	2.16 (0.98, 4.47)	0.06
*p* for trend	–	–		0.33		0.33		0.93
**Dosage density (μg/day)**								
0	67	19 (28.36%)	1		1		1	
< 15	71	34 (47.89%)	1.60 (0.91, 2.82)	0.10	1.65 (0.93, 2.92)	0.05	1.54 (0.82, 2.90)	0.18
15–60	89	38 (42.70%)	1.76 (1.01, 3.06)	0.04	1.80 (1.03, 3.14)	0.04	1.50 (0.81, 2.81)	0.20
60–200	38	20 (52.63%)	2.45 (1.30, 4.60)	< 0.01	2.73 (1.44, 5.18)	0.01	2.07 (1.04, 4.09)	0.04
≥ 200	42	24 (57.14%)	3.89 (2.12, 7.12)	< 0.01	3.80 (2.06, 7.01)	< 0.01	3.36 (1.77, 6.37)	< 0.001
*p* for trend				< 0.001		< 0.001		< 0.001

Non-adjusted model adjust for: None

Adjusted I model adjust for: Age, Sex, Weight, Smoking

Adjusted II model adjust for: Age, Sex, Weight, Smoking, Histopathology, Differentiation, Complications, Pathological stage, Chemotherapy, Number of chemotherapy, Myelosuppression, Fever, Antibiotic

*HR* Hazard ratio, *CI* Confidence interval, *rhG-CSF* Recombinant human granulocyte-colony stimulating factor

**Table 4:** Effect of any rhG-CSF use on metastasis following postoperative chemotherapy in NSCLC by follow-up 1, 2 and 3 year

	Follow-up 1 Year				Follow-up 2 Year			Follow-up 3 Year		
	Total, n	Events, n (%)	Adjusted I	Adjusted II	Events, n (%)	Adjusted I	Adjust II	Events, n (%)	Adjusted I	Adjusted II
			HR (95% CI)	HR (95% CI)		HR (95% CI)	HR (95% CI)		HR (95% CI)	HR (95% CI)
**rhG-CSF**										
No rhG-CSF	61	4 (6.56%)	1	1	8 (13.11%)	1	1	12 (19.67%)	1	1
rhG-CSF	246	54 (21.95%)	4.02 (1.45,11.11)	4.44 (1.54,12.79)	80 (32.52%)	3.17 (1.53, 6.58)	2.91 (1.34, 6.29)	101 (41.05%)	2.66 (1.45, 4.86)	2.53 (1.32, 4.82)
**Doseage (μg)**										
0	61	4 (6.56%)	1	1	8 (13.11%)	1	1	12 (19.67%)	1	1
≤ 500	96	19 (19.79%)	3.74 (1.27, 11.01)	4.09 (1.36, 12.34)	32 (33.33%)	3.37 (1.55, 7.33)	3.13 (1.40, 7.00)	41 (42.71%)	2.91 (1.52, 5.57)	2.76 (1.41, 5.43)
500–1000	85	20 (23.53%)	4.36 (1.49, 12.82)	5.36 (1.70, 16.89)	26 (30.59%)	3.00 (1.35, 6.65)	2.79 (1.18, 6.64)	32 (37.65%)	2.47 (1.29, 4.82)	2.50 (1.20, 5.20)
1000–1500	32	6 (18.75%)	2.97 (0.84, 10.58)	3.57 (0.96, 13.27)	10 (31.25%)	2.53 (0.99, 6.47)	2.30 (0.87, 6.06)	13 (40.63%)	2.06 (0.93, 4.57)	1.88 (0.82, 4.31)
> 1500	33	9 (27.27%)	5.21 (1.59, 17.01)	7.18 (1.96, 26.26)	12 (36.36%)	3.88 (1.58, 9.54)	3.88 (1.42, 10.59)	15 (45.45%)	3.18 (1.48, 6.82)	3.12 (1.32, 7.38)
*p* for trend			0.04	0.03		0.05	0.16		0.08	0.23
**Dose density (μg/day)**										
0	67	5 (7.46%)	1	1	10 (14.93%)	1	1	15 (22.39%)	1	1
< 15	71	13 (18.31%)	2.52 (0.90, 7.08)	2.91 (0.97, 8.75)	20 (28.17%)	1.97 (0.92, 4.23)	1.89 (0.83, 4.3)	29 (40.85%)	1.83 (0.98, 3.42)	1.79 (0.90, 3.55)
15–60	89	21 (13.16%)	3.65 (1.37, 9.71)	3.74 (1.31, 10.67)	28 (31.46%)	2.63 (1.27, 5.42)	2.16 (0.98, 4.76)	31 (34.83%)	1.87 (1.01, 3.48)	1.59 (0.80, 3.15)
60–200	38	5 (13.16%)	2.25 (0.65, 7.84)	1.97 (0.55, 7.15)	11 (28.95%)	2.48 (1.05, 5.88)	1.83 (0.74, 4.51)	16 (42.11%)	2.51 (1.23, 5.12)	1.88 (0.89, 3.97)
≥ 200	42	14 (33.33%)	6.13 (2.19, 17.11)	5.88 (2.06, 16.85)	19 (45.24%)	4.97 (2.3, 10.75)	4.18 (1.88, 9.29)	22 (52.38%)	4.09 (2.11, 7.94)	3.38 (1.69, 6.74)
*p* for trend			< 0.001	0.01		< 0.001	< 0.001		< 0.001	< 0.001

Adjusted I model adjust for: Age; Sex; Weight; Smoking

Adjusted II model adjust for: Age; Sex; Weight; Smoking; Histopathology; Differentiation; Complications; Pathological stage; Chemotherapy; Number of chemotherapy; Myelosuppression; Fever; Antibiotic

*HR* Hazard ratio, *CI* Confidence interval, *rhG-CSF* Recombinant human granulocyte-colony stimulating factor

### Subgroup analyses by important covariables

**Fig. 3:**
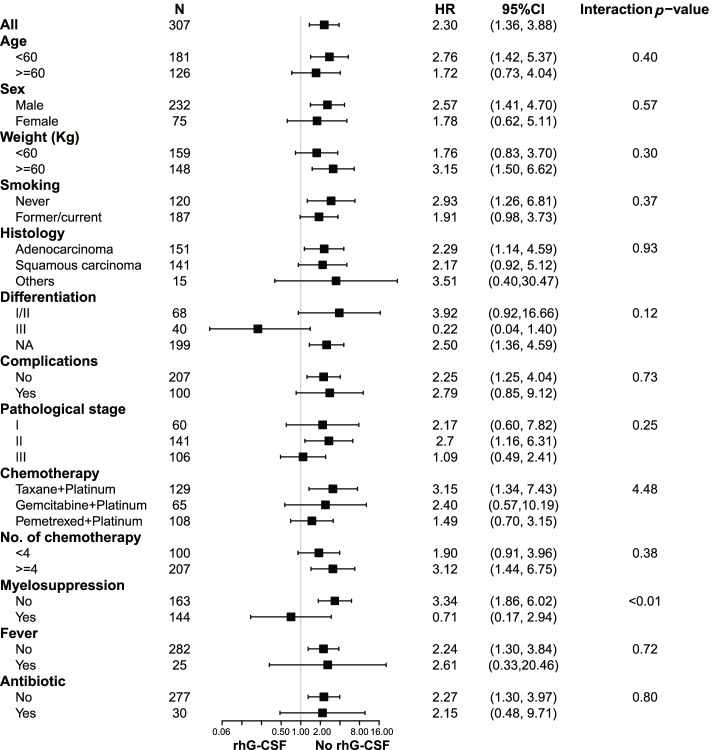
Subgroup analysis on effect of rhG-CSF on tumor metastasis in postoperative NSCLC patients accepted chemotherapy

**Fig. 4:**
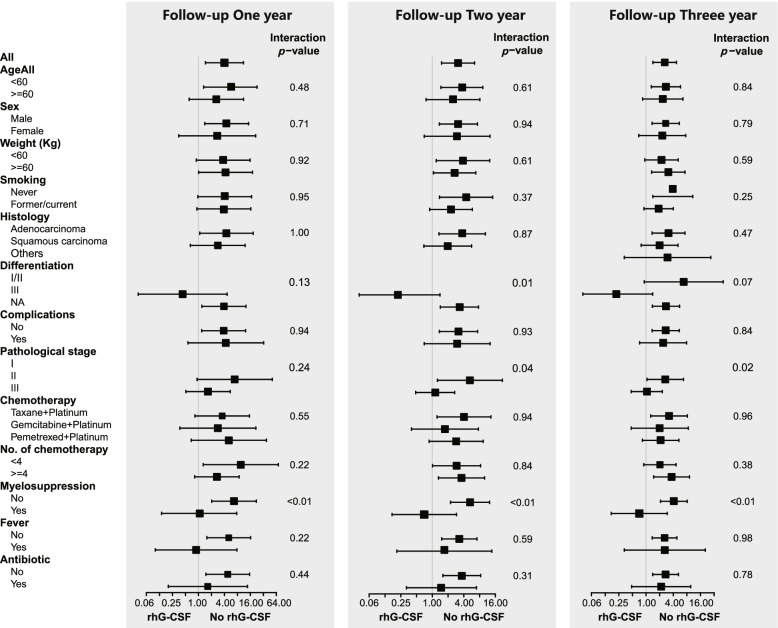
Subgroup analyses on effect of rhG-CSF on tumor metastasis in postoperative NSCLC patients accepted chemotherapy during different follow-up times. **A**-**C** Follow-up postoperative NSCLC patients accepted chemotherapy 1, 2, and 3 years

To further confirm the results that rhG-CSF was an independent adverse factor of metastasis that was robust to potential confounders, we performed subgroup analyses of major covariables that may be related to metastasis, including age, sex, weight, smoking, histology, differentiation, complications, pathological stage, chemotherapy regimen, number of chemotherapies, myelosuppression, fever, and antibiotics. Subgroup analysis showed a similarly consistent pattern (Fig. 3), and there were no significant interactions in most of the covariables (interaction *p*-value> 0.05) except for myelosuppression (interaction *p*-value< 0.01). Among patients who presented without myelosuppression after chemotherapy, rhG-CSF treatment resulted in a significantly high risk of metastasis (HR 3.34, 95% CI 1.86–6.02, *p* < 0.01). In contrast, rhG-CSF treatment reduced the risk of metastasis in patients who presented myelosuppression (HR 0.71, 95% CI 0.17–2.94, *p* > 0.05). Compared to the patients who had myelosuppression after chemotherapy, patients who presented without myelosuppression had a higher risk correlation of rhG-CSF use with metastasis (HR 3.34 vs. 0.71, interaction *p*-value< 0.01), and at postoperative follow-up 1 year (HR 6.67 vs. 1.05, interaction *p*-value< 0.01), 2 years (HR 5.26 vs. 0.70, interaction *p*-value< 0.01) and 3 years (HR 4.11 vs. 0.71, interaction *p*-value< 0.01) (Fig. 4).

### Effects of dose and density of rhG-CSF treatment on the risk of metastasis

We further examined the degree to which rhG-CSF increased the risk of metastasis in various dose gradient ranges. We observed an increased risk of metastasis with rhG-CSF use at different doses, except for the 1000 μg–1500 μg gradient group (*p* > 0.05), in the non-adjusted and adjusted models (Table 3). However, the risk of metastasis did not increase as the dose of rhG-CSF increased (*p* for trend> 0.05). When the factor of time was considered, the dose density of rhG-CSF, which is dose of rhG-CSF used in the length of rhG-CSF exposure time, a positive relationship between the dose density of rhG-CSF treatment and risk of metastasis was observed for all models. Statistically significant results of trend tests were obtained in the total sample (*p* for trend< 0.001) (Table 3). The analogous consequences were exhibited in the postoperative follow-up at 1, 2, and 3 years (*p* for trend< 0.05) (Table 4).

## Discussion

Distant organ metastasis is always associated with poor prognosis and shorter survival in NSCLC patients [13]. The specific adverse factors for DOM in NSCLC patients with postoperative chemotherapy are not entirely clear. To the best of our knowledge, the current study is the first retrospective cohort study to elucidate the effect of rhG-CSF on cancer metastasis in NSCLC patients with postoperative chemotherapy. The median time to postoperative metastasis of NSCLC patients was 37.23 months in our study, which is consistent with NSCLC patients in China [14]. Consistent with routine clinical medication [15], our research demonstrated that rhG-CSF was commonly used in NSCLC patients with multiple chemotherapy treatments and myelosuppression. Therefore, the trend that we observed was justifiable.

The influence of tumour staging on patient metastasis has been generally recognized [16]. For pathophysiology, a sizeable solid piece of lung cancer could generate large metastatic lymph nodes to enhance metastasis risk [17, 18]. High G-CSF levels within tumour tissue may represent a high risk of DOM and a worse prognosis [19, 20]. As found in our present study, these two adverse factors were also related to DOM occurrence. Poor nutrition conditions are a powerful underlying prognosticator of postoperative DOM [16, 21]. To avoid the confounding factors as much as possible, we removed patients with PS scores ≥ 2 before postoperative chemotherapy in the analysis. The total dose of rhG-CSF used was influenced by the number of chemotherapies, chemotherapy regimens, and the degree of myelosuppression. Therefore, chemotherapy cycle, regimens, and myelosuppression status were also adjusted in the analysis. After taking these potential factors into account, the results suggested that rhG-CSF had an intimate connection with DOM following postoperative chemotherapy. Because the loss to follow-up may result in withdrawal bias, it also likely distorted the results because the proportion of patients who were lost to follow-up increased with the follow-up time. We also analysed the correlation between rhG-CSF treatment and DOM based on postoperative follow-up at 1, 2, and 3 years. The outcomes showed that the risk of DOM was significantly higher than the No rhG-CSF group at 1, 2, or 3 years of follow-up after surgery. These results suggest that rhG-CSF was connected with DOM in this condition, which offers persuasive evidence for our study.

CSF was first discovered by Don Metcalf et al. [22]. Mice treated with rhG-CSF had a high level of granulocytes in the spleen [23], which led to the wide use of rhG-CSF in clinical practice to prevent the development of FN in patients undergoing intensive chemotherapy that induced myelosuppression. Patients with myelosuppression following chemotherapy exhibit neutropenia, efficient induction of FN or lethal infection. RhG-CSF treatment supplementation plays a vital role in allowing patients to safely accomplish a myelosuppression-toxic chemotherapy regimen, which improves the disease-free survival (DFS) and OS of patients after chemotherapy [6]. Notably, the use of rhG-CSF in the clinic has increased over the past 30 years [6]. From our study, 80.13% of patients had been injected with rhG-CSF at a mean dose of 885.89 μg. Regrettably, our study revealed that rhG-CSF injection during postoperative chemotherapy was related to the occurrence of DOM. The data further suggested that the risk of DOM increased with increasing rhG-CSF dose density. These results also suggest that a more precise standard is needed to direct the use of rhG-CSF to reduce the risk of DOM.

Current guidelines for rhG-CSF of National Comprehensive Cancer Network (NCCN) treatment recommend prophylaxis with rhG-CSF when the risk of FN is approximately 20% or higher or the risk of FN ranges from 10 to 20% combined with one or more risk factors, such as age and recent history of surgery [24]. The purpose of prophylaxis with rhG-CSF is to prevent or remit the degree of neutropenia after chemotherapy or shorten the time of neutropenia to lower the risk of FN, severe infection, and death. As the guidelines introduced [24], no clear evidence suggests that the therapeutic use of rhG-CSF provides outstanding therapeutic value for patients who have had severe infection or neutropenia without fever compared to the prophylactic use of rhG-CSF. However, how many patients will have infections due to neutropenia after chemotherapy because they do not use rhG-CSF treatment in the real world is not known. Even if a certain degree of neutropenia occurs, it may not be many patients who experience the events that the guidelines focus on. A prospective, phase III, randomised, double-blind, multicentre study indicated that the FN rate was 5.6% in the placebo (*n* = 125) group during cycle 1 of chemotherapy in NSCLC patients [25]. Zhou et al. [26] found that only 8% (*n* = 51) of patients with NSCLC who received chemotherapy without rhG-CSF treatment exhibited FN. There was a relatively low occurrence of FN in NSCLC patients even without rhG-CSF. It was evident that a large proportion of patients who received mild chemotherapy could also safely survive the risk period after chemotherapy in the absence of rhG-CSF. Guidelines pay close attention to whether the use of rhG-CSF reduces the occurrence of FN and its complications, the risk of infection and death, the duration of severe neutropenia, the duration of antibiotic use, the length of hospital stay, bone pain, and other indicators. However, it may ignore the dark side of rhG-CSF supplements, such as promoting cancer metastasis. The risk of metastasis with excessive use of rhG-CSF was increased remarkably in most of these patients, which led us to consider whether certain precautions, such as avoiding some infection factors and increasing nutrition after chemotherapy, could take the place of rhG-CSF use to lower the risk. Although patients with breast cancer benefitted from rhG-CSF use in extending DFS and OS, any damage from rhG-CSF use could be whitewashed by the profit supported by dose-intensity chemotherapy regimens [27]. However, chemotherapy regimens for NSCLC in the guidelines are mostly moderate-risk strategies, unlike the high FN risk induced by chemotherapy for breast cancer [24].

A developing number of studies [28–30] identified that rhG-CSF promoted primary tumour growth and metastasis in preclinical experiments. In a Lewis lung carcinoma mouse model, rhG-CSF combined with paclitaxel (PTX) stimulated angiogenesis and subsequent tumour regrowth, which minimised the effect of chemotherapy [8]. Few positive results have been revealed in clinical studies. The reason lies in rhG-CSF being injected before or very soon after building tumour animal models [7]. This gives full play to the rhG-CSF character in promoting metastasis. However, in clinical treatment, diagnosed patients received rhG-CSF after chemotherapy. It is possible that the pro-metastatic effect of rhG-CSF is exerted in a limited time or that the benefits of chemotherapy would overcome the disadvantages of rhG-CSF. Similarly, our research determined that the function of rhG-CSF in promoting metastasis relied on dose density rather than the total dose. Therefore, using rhG-CSF frequently in a short time puts patients at high risk of metastasis and worsens prognosis. It also prompted us to think deeply about whether we should avoid high-frequency rhG-CSF use in a short time during clinical practice. However, the benefits of rhG-CSF are apparent but are accompanied by an increased risk of DOM.

Neutrophil extracellular traps (NETs) are a network structure consisting of modified chromatin structures and decorated with given cytoplasmic and granular proteins, which likely also played a crucial part in the enhanced risk of DOM with rhG-CSF use [31]. NETs, first identified by Brinkmann et al. in 2004 [32], are released when neutrophils trap and kill pathogenic microorganisms and activate platelets [33]. Notably, Tohme et al. discovered that serum NET levels of patients undergoing hepatectomy were markedly elevated in CRC patients, which distinguished them from healthy controls, and had a greater risk of poor prognosis [34]. Abundant studies suggested that G-CSF aggravated NET formation in preclinical tumour experiments [31, 35, 36]. Unexpectedly, in contrast to myelosuppression, rhG-CSF was significantly associated with DOM in NSCLC patients without myelosuppression in the results of our analyses. We hypothesise that rhG-CSF injection primarily elevates the level of NETs under non-myelosuppression circumstances, which encourages DOM in the mechanism and validates it in the future. As one of the most common manifestations of infection, fever may be associated with the appearance of NETs. Therefore, the risk of DOM may be boosted following fever and reduced by antibiotic treatment. Regrettably, our results do not reflect the hypothesis we proposed, perhaps due to the small sample size. Therefore, more samples to validate the proposed hypothesis are needed for future analyses.

The analyses of this research offer proof of the effect of rhG-CSF on the development of DOM in NSCLC patients with postoperative chemotherapy. Therefore, a physician in clinical practice should administer rhG-CSF to NSCLC patients based on chemotherapy regimens and the patient’s own risk factors using rhG-CSF once myelosuppression exists. The recommended guidelines for rhG-CSF use should formulate more comprehensive standards. Populations that are suitable to use rhG-CSF are also worthy of further exploration.

Our study has the following limitations. First, due to loss to follow-up, we did not dissect the OS of participants in this study, which should be shown in subsequent research. Second, our study only included NSCLC patients who received chemotherapy postoperatively. Future studies should consider more tumour types. DOM was primarily judged using imaging examination and not biopsy, which likely caused false-positive results, despite the diagnosis estimated by two radiologists. Pathological discoveries may be used to supply evidence microenvironment variations in the metastatic organs. Lastly, this study was a retrospective study, and some selection biases may be present. Prospective research is also needed to verify these conclusion.

## Conclusion

Among NSCLS patients following postoperative chemotherapy, we found that rhG-CSF use was related to a high risk of metastasis, and this risk may increase significantly with the increase in dose density of rhG-CSF. The study suggests that patients without myelosuppression have more disadvantages from rhG-CSF. Our findings, if affirmed, lead to far-reaching clinical and public health influence because of the highest mortality rate of NSCLC globally. This result suggests that identifying patients with or without myelosuppression after chemotherapy could help guide the rational drug use of rhG-CSF and avoid its risks in clinical use.

## Data Availability

This manuscript included all data collected or analyzed during this research.

## Abbreviations

G-CSF: Granulocyte colony-stimulating factor
rhG-CSF: Recombinant human granulocyte colony-stimulating factor
NSCLC: Non-small cell lung cancer
DOM: Distant organ metastasis
FN: Febrile neutropenia
OS: Overall survival
HNSCC: Head and neck squamous cell carcinoma
CTCAE: Common Terminology Criteria for Adverse Events
US: Ultrasound
CT: Computed tomography
MRI: Magnetic resonance imaging
ECT: Emission computed tomography
MFS: Metastasis-free survival
ECOG PS: Eastern Cooperative Oncology Group performance status
DFS: Disease-free survival
NCCN: National Comprehensive Cancer Network
PTX: Paclitaxel
NETs: Neutrophil extracellular traps
CRC: Colorectal cancer

## References

[1] Siegel RL, Miller KD, Jemal A (2019). Cancer statistics, 2019. CA Cancer J Clin.

[2] Ekeke CN, Mitchell C, Schuchert M, Dhupar R, Luketich JD, Okusanya OT (2021). Early distant recurrence in patients with resected stage I lung cancer: a case series of “blast metastasis”. Clin Lung Cancer.

[3] Chen Y, Cai C, Li Y (2021). The impact of baseline brain metastases on clinical benefits and progression patterns after first-line crizotinib in anaplastic lymphoma kinase-rearranged non-small cell lung cancer. Medicine (Baltimore).

[4] Chen VW, Ruiz BA, Hsieh MC, Wu XC, Ries LA, Lewis DR (2014). Analysis of stage and clinical/prognostic factors for lung cancer from SEER registries: AJCC staging and collaborative stage data collection system. Cancer.

[5] Qiu Z, Ye B, Zhao S, Li X, Li L, Mo X (2019). Non-canonical Raf-1/p70S6K signalling in non-small-cell lung cancer. J Cell Mol Med.

[6] Lyman GH, Reiner M, Morrow PK, Crawford J (2015). The effect of filgrastim or pegfilgrastim on survival outcomes of patients with cancer receiving myelosuppressive chemotherapy. Ann Oncol.

[7] Yeo B, Redfern AD, Mouchemore KA, Hamilton JA, Anderson RL (2018). The dark side of granulocyte-colony stimulating factor: a supportive therapy with potential to promote tumour progression. Clin Exp Metastasis.

[8] Kowanetz M, Wu X, Lee J, Tan M, Hagenbeek T, Qu X (2010). Granulocyte-colony stimulating factor promotes lung metastasis through mobilization of Ly6G+Ly6C+ granulocytes. Proc Natl Acad Sci U S A.

[9] Swierczak A, Cook AD, Lenzo JC, Restall CM, Doherty JP, Anderson RL (2014). The promotion of breast cancer metastasis caused by inhibition of CSF-1R/CSF-1 signaling is blocked by targeting the G-CSF receptor. Cancer Immunol Res.

[10] Kumar J, Fraser FW, Riley C, Ahmed N, McCulloch DR, Ward AC (2014). Granulocyte colony-stimulating factor receptor signalling via Janus kinase 2/signal transducer and activator of transcription 3 in ovarian cancer. Br J Cancer.

[11] Gutschalk CM, Herold-Mende CC, Fusenig NE, Mueller MM (2006). Granulocyte colony-stimulating factor and granulocyte-macrophage colony-stimulating factor promote malignant growth of cells from head and neck squamous cell carcinomas in vivo. Cancer Res.

[12] Chao D, Hu G, Li Q (2021). Clinicopathological significance and prognostic value of E-cadherin expression in non-small cell lung cancer: a protocol for systematic review and meta-analysis. Medicine (Baltimore).

[13] Black RC, Khurshid H (2015). NSCLC: an update of driver mutations, their role in pathogenesis and clinical significance. R I Med J (2013).

[14] Zhu Z, Chai Y (2021). First-line EGFR-TKIs treatment in stage I non-small-cell lung cancer patients harboring EGFR gene mutations with postoperative intrapulmonary recurrence. Cancer Manag Res.

[15] Fujita A, Ohkubo T, Hoshino H, Takabatake H, Tagaki S, Sekine K (2003). Phase II study of cisplatin, ifosfamide, and irinotecan with rhG-CSF support in patients with stage IIIb and IV non-small-cell lung cancer. Br J Cancer.

[16] Lin M, Chen QY, Zheng CH, Li P, Xie JW, Wang JB (2021). Effect of preoperative tumour under-staging on the long-term survival of patients undergoing radical gastrectomy for gastric cancer. Cancer Res Treat.

[17] Zhang F, Zheng W, Ying L, Wu J, Wu S, Ma S (2016). A nomogram to predict brain metastases of resected non-small cell lung cancer patients. Ann Surg Oncol.

[18] Cho JY, Leem CS, Kim Y, Kim ES, Lee SH, Lee YJ (2018). Solid part size is an important predictor of nodal metastasis in lung cancer with a subsolid tumor. BMC Pulm Med.

[19] Hollmén M, Karaman S, Schwager S, Lisibach A, Christiansen AJ, Maksimow M (2016). G-CSF regulates macrophage phenotype and associates with poor overall survival in human triple-negative breast cancer. Oncoimmunology.

[20] Welte T, Kim IS, Tian L, Gao X, Wang H, Li J (2016). Oncogenic mTOR signalling recruits myeloid-derived suppressor cells to promote tumour initiation. Nat Cell Biol.

[21] Takada K, Shimokawa M, Akamine T, Ono Y, Haro A, Osoegawa A (2019). Association of low body mass index with poor clinical outcomes after resection of non-small cell lung cancer. Anticancer Res.

[22] Burgess AW, Metcalf D (1980). The nature and action of granulocyte-macrophage colony stimulating factors. Blood.

[23] Fujisawa M, Kobayashi Y, Okabe T, Takaku F, Komatsu Y, Itoh S (1986). Recombinant human granulocyte colony-stimulating factor induces granulocytosis in vivo. Jpn J Cancer Res.

[24] Crawford J, Becker PS, Armitage JO, Blayney DW, Chavez J, Curtin P (2017). Myeloid growth factors, version 2.2017, NCCN clinical practice guidelines in oncology. J Natl Compr Cancer Netw.

[25] Volovat C, Bondarenko IM, Gladkov OA, Elsässer R, Buchner A, Bias P (2015). Phase III, randomized, double-blind, placebo-controlled, multicenter study of lipegfilgrastim in patients with non-small cell lung cancer receiving myelosuppressive therapy. Springerplus.

[26] Zhou C, Huang Y, Wang D, An C, Zhou F, Li Y (2016). A randomized multicenter phase III study of single administration of mecapegfilgrastim (HHPG-19K), a pegfilgrastim biosimilar, for prophylaxis of chemotherapy-induced neutropenia in patients with advanced non-small-cell lung cancer (NSCLC). Clin Lung Cancer.

[27] Han Y, Yu Z, Wen S, Zhang B, Cao X, Wang X (2012). Prognostic value of chemotherapy-induced neutropenia in early-stage breast cancer. Breast Cancer Res Treat.

[28] Hamilton JA, Cook AD, Tak PP (2016). Anti-colony-stimulating factor therapies for inflammatory and autoimmune diseases. Nat Rev Drug Discov.

[29] Morris KT, Khan H, Ahmad A, Weston LL, Nofchissey RA, Pinchuk IV (2014). G-CSF and G-CSFR are highly expressed in human gastric and colon cancers and promote carcinoma cell proliferation and migration. Br J Cancer.

[30] Wang J, Yao L, Zhao S, Zhang X, Yin J, Zhang Y (2012). Granulocyte-colony stimulating factor promotes proliferation, migration and invasion in glioma cells. Cancer Biol Ther.

[31] Demers M, Wong SL, Martinod K, Gallant M, Cabral JE, Wang Y (2016). Priming of neutrophils toward NETosis promotes tumor growth. Oncoimmunology.

[32] Brinkmann V, Reichard U, Goosmann C, Fauler B, Uhlemann Y, Weiss DS (2004). Neutrophil extracellular traps kill bacteria. Science.

[33] Branzk N, Papayannopoulos V (2013). Molecular mechanisms regulating NETosis in infection and disease. Semin Immunopathol.

[34] Tohme S, Yazdani HO, Al-Khafaji AB, Chidi AP, Loughran P, Mowen K (2016). Neutrophil extracellular traps promote the development and progression of liver metastases after surgical stress. Cancer Res.

[35] Xu D, Lin Y, Shen J, Zhang J, Wang J, Zhang Y (2020). Overproduced bone marrow neutrophils in collagen-induced arthritis are primed for NETosis: an ignored pathological cell involving inflammatory arthritis. Cell Prolif.

[36] Arpinati L, Shaul ME, Kaisar-Iluz N, Mali S, Mahroum S, Fridlender ZG (2020). NETosis in cancer: a critical analysis of the impact of cancer on neutrophil extracellular trap (NET) release in lung cancer patients vs. mice. Cancer Immunol Immunother.

